# Umbilical hernia in autosomal dominant polycystic kidney disease

**DOI:** 10.1007/s10157-013-0927-0

**Published:** 2014-01-10

**Authors:** Yoshinari Hattori, Junichi Hoshino, Tatsuya Suwabe, Keiichi Sumida, Koki Mise, Noriko Hayami, Naoki Sawa, Kenmei Takaichi, Yoshifumi Ubara

**Affiliations:** 1 Nephrology Center, Toranomon Hospital, 2-2-2 Toranomon, Minato, Tokyo, 105-8470 Japan; 2 Okinaka Memorial Institute for Medical Research, Toranomon Hospital, Tokyo, Japan

**Keywords:** Umbilical hernia, Autosomal dominant polycystic kidney disease

**Fig. 1:**
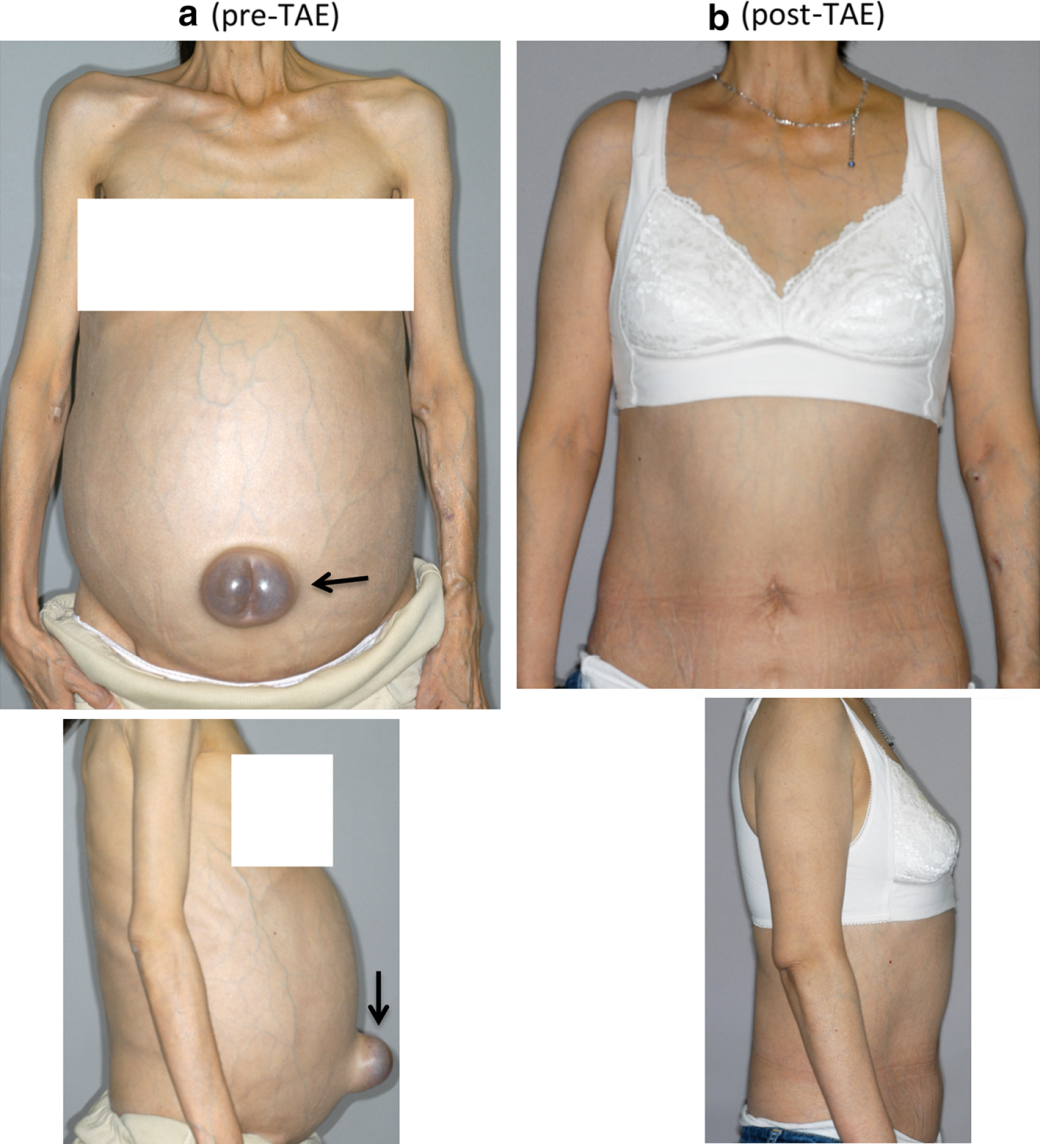
**a** Gross appearance of pre-TAE. **b** Gross appearance of post-TAE. *Arrow* shows protruded umbilical hernia

**Fig. 2:**
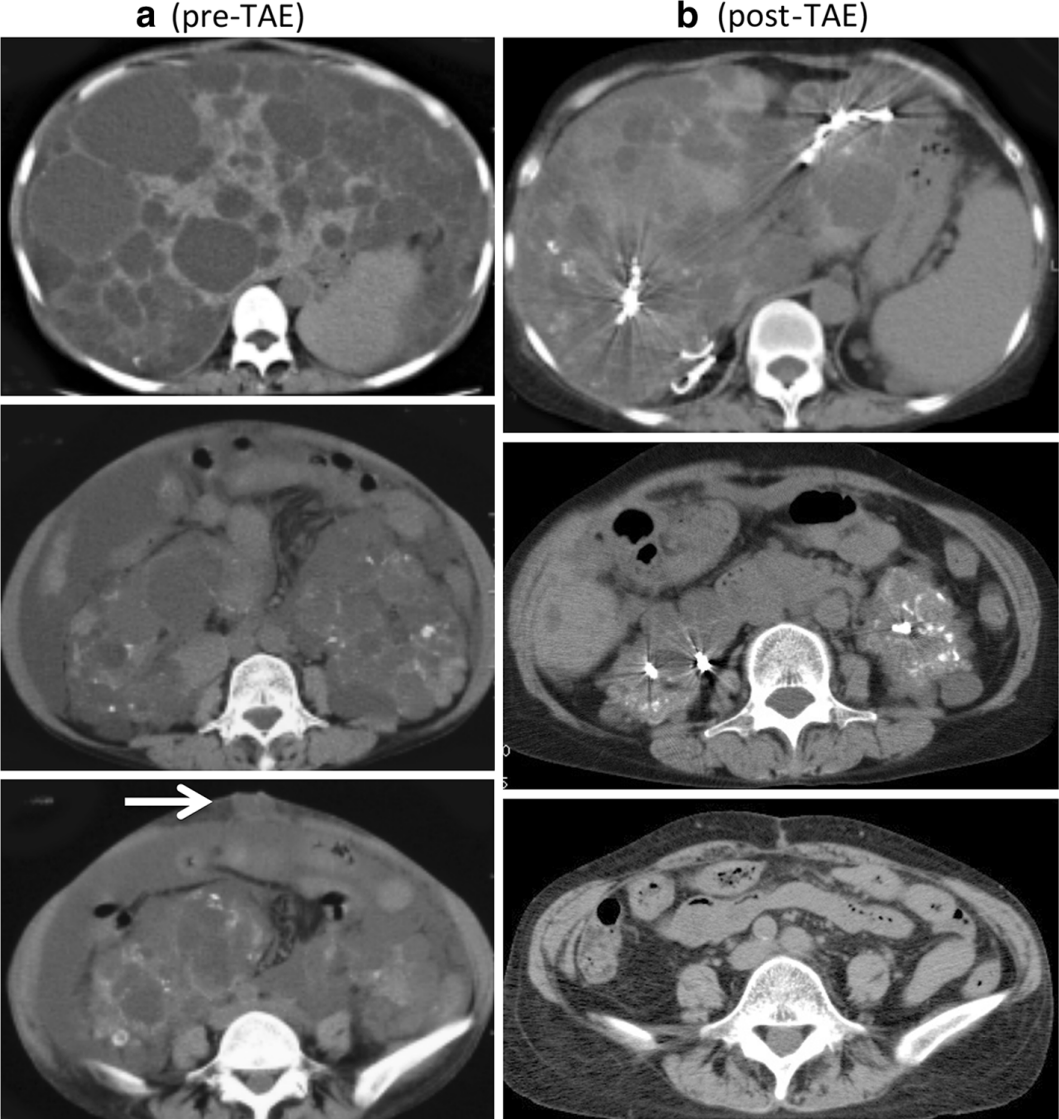
**a** Computed tomography images pre-TAE. **b** Computed tomography images post-TAE. *Arrow* shows protruded umbilical hernia

A 44-year-old woman was diagnosed with autosomal dominant polycystic kidney disease. Her mother has the same disease. Even after hemodialysis was started in 2003 due to end-stage renal failure, abdominal distention progressed and a protruding umbilical hernia became prominent (Fig. 1a, b). However, the surgeons hesitated to perform hernia repair. Transcatheter arterial embolization (TAE) was performed to treat massive hepatomegaly in 2005 [1] and to treat bilateral nephromegaly in 2006 [2]. Her abdominal distension and umbilical hernia both improved in 2013 (Fig. 2a, b). This case emphasizes that massive polycystic liver and kidneys may contribute to umbilical hernia formation by increasing the intra-abdominal pressure.
